# Residual pain and fatigue are affected by disease perception in rheumatoid arthritis in sustained clinical and ultrasound remission

**DOI:** 10.1007/s10067-025-07331-0

**Published:** 2025-01-22

**Authors:** Simone Perniola, Dario Bruno, Clara Di Mario, Denise Campobasso, Martina Calabretta, Marco Gessi, Luca Petricca, Barbara Tolusso, Stefano Alivernini, Elisa Gremese

**Affiliations:** 1 Immunology Research Core Facility, Gemelli Science and Technology Park (GSTeP), Fondazione Policlinico Universitario A. Gemelli IRCCS, Rome, Italy; 2 Clinical Immunology Division, Fondazione Policlinico Universitario A. Gemelli IRCCS, Rome, Italy; 3 Rheumatology Division, Fondazione Policlinico Universitario A. Gemelli IRCCS, Rome, Italy; 4 Pathology Institute, Fondazione Policlinico Universitario A. Gemelli IRCCS, Rome, Italy; 5 Università Cattolica del Sacro Cuore, Rome, Italy; 6 Medicine Department, Università Degli Studi Di Verona, Verona, Italy

**Keywords:** Pain, Patient-reported outcomes, Remission, Rheumatoid arthritis

## Abstract

**Objective:**

Regardless of remission status, residual pain (RP) might persist in rheumatoid arthritis (RA). The aim of this study was to characterize RP, its perception, and patient-dependent features and to evaluate its possible association with residual synovitis in patients with RA in remission.

**Methods:**

Ninety-seven patients with RA, including 68 in sustained clinical and ultrasound remission (Rem/RA) and 29 in high/moderate DAS28-CRP disease activity (H-Mo/RA) were enrolled in the study. Thirty patients with fibromyalgia were enrolled as a control group(FIBRO). At study entry, demographic, clinical, ultrasound characteristics, and pain dimension assessment (VAS-pain, FACIT, CSI, GHQ, and RAID) were collected for each patient. RA patients underwent synovial tissue biopsy to evaluate the degree of synovitis using the Krenn synovitis score (KSS).

**Results:**

Forty-eight percent of Rem/RA still declared unacceptable pain (VAS-Pain > 20) compared to 80% of H-Mo/RA patients (*p* < 0.0001). Furthermore, Rem/RA patients presented comparable levels of pain dimension assessment regardless of KSS. However, classifying Rem/RA group based on RAID score (< 2 as satisfied SAT-Rem/RA and ≥ 2 as unsatisfied UNSAT-Rem/RA), SAT-Rem/RA group presented a lower grade of VAS-Pain (*p* < 0.0001), lower percentage of patients with an unacceptable pain (*p* < 0.0001) and lower grade of fatigue(*p* < 0.0001) compared to the UNSAT-Rem/RA patients. The percentage of SAT-Rem/RA patients who presented a disease flare did not differ from UNSAT-Rem/RA over the 24 months of follow-up. Finally, female Rem/RA patients presented higher VAS-Pain compared to male Rem/RA (*p* = 0.0119).

**Conclusions:**

Moreover,73% satisfied female Rem/Ra patients presented an acceptable pain compared to 23% unsatisfied female Rem/RA patients (*p* = 0.001). RP in RA patients in remission can represent the way by which the patients communicate their state of non-acceptance of the disease. It can be useful to treat RP with the appropriate treatments.

**Table:** 

**Key Points** • *Rheumatoid arthritis patients still reported unacceptable residual pain despite sustained clinical and ultrasound remission and despite the low grade/absence of histological synovitis.* • *Only a small rate of rheumatoid arthritis patients in sustained clinical and ultrasound remission showed residual pain as part of a central sensitivity syndrome or psychiatric disorders.* • *Rheumatoid arthritis patients in sustained clinical and ultrasound remission complained residual pain and fatigue as part of not acceptance of disease and/or dissatisfaction in the disease management.*

**Supplementary Information:**

The online version contains supplementary material available at 10.1007/s10067-025-07331-0.

## Introduction

To date, the primary goal in the management of rheumatoid arthritis (RA) is the achieving of sustained remission [1], which significantly reduces the risk of radiographic progression and disability. Furthermore, remission is associated with a better control of the symptoms, including a notable reduction in perceived pain [2]. This objective is widely endorsed across clinical guidelines, including those issued by the American College of Rheumatology (ACR) and the European Alliance of Associations for Rheumatology (EULAR), reflecting its global importance [1, 3].

Pain is described as an unpleasant sensation and can be classified as nociceptive, neuropathic, or nociplastic, based on its causes [4]. RA pain is predominantly nociceptive, resulting from inflammatory processes that primarily affect joint structures. However, some patients also report nociplastic pain (in terms of central sensitization disorders), which is characterized by an abnormal response to stimuli capable of triggering pain [5, 6]. Regardless of its origin, emotional and cultural elements can impact pain perception, making it increasingly important to use specific tools that accurately quantify and interpret the nature and extent of pain [7, 8]. Understanding the nature of pain is crucial for the proper management of RA, even during its remission stages. In particular, nociplastic pain does not respond to conventional treatments typically used for nociceptive pain, such as analgesics and anti-inflammatory drugs [9]. Furthermore, in osteoarthritis, neuropathic/nociplastic pain serves as an independent prognostic factor for knee prosthetic replacement failure [10].

In RA, residual pain (RP) is defined as the persistence of pain despite the achievement of the low disease activity (LDA) or the remission status [11]. Different studies reported that from 9 to 50% of RA patients in LDA or in various definitions of remission complained about RP and fatigue [11–15]. RP and fatigue may affect the patient daily activity and quality of life not only causing discomfort but sometimes placing additional burden such as causing sleep disorders, even in remission or low disease activity status [16].

RA in clinical and ultrasound remission can show persistent histological residual synovitis with immune cell infiltration at the synovial tissue level [17–19], and this might be involved in the pathophysiology of RP.

Although RP in RA has been documented, little is known about the extent to which nociceptive and nociplastic mechanisms contribute to RP, how synovitis degree impacts RP, and how patients’ perception of the disease influences their pain experience.

The aims of this study were (i) to characterize the extent of RP and its perception in RA patients in sustained clinical and ultrasound remission compared to RA patients with active disease and patients with fibromyalgia and (ii) to assess its association with patients’ characteristics and residual synovitis histological degree.

## Patients and methods

### Patient selection

Ninety-seven consecutive RA patients fulfilling the American College of Rheumatology/European League Against Rheumatism (ACR/EULAR) 2010 classification criteria [20], not affected by concomitant fibromyalgia (RA group), and 30 consecutive patients fulfilling the 2016 ACR criteria for fibromyalgia [21] as control group (FIBRO) were enrolled at the Fondazione Policlinico Universitario A. Gemelli IRCCS in Rome, Italy. At study entry, clinical and demographic characteristics were collected, and a comprehensive disease activity assessment was performed for each enrolled patient. In particular, the following parameters were collected for each RA patient: erythrosedimentation rate (ESR) and C-reactive protein (CRP) plasma levels, body mass index (BMI), anti-cyclic citrullinated protein antibodies (ACPA) and rheumatoid factor (RF) status, and CRP-based disease activity score on 28 joints (DAS28-CRP), bone erosive status assessed by plain X-Rays of hands, wrists, ankles, and feet bilaterally, and the concomitant use of corticosteroids, csDMARDs, and/or bDMARDs.

Patients provided their written informed consent to take part with explicit protection of identification. This study complies with the ethical guidelines of the 1975 Helsinki Declaration and was approved by the local Ethics Committee (protocol no. 28485/18 for the SYNGem cohort).

### Management of RA patients

RA patients were divided into two groups based on DAS28-CRP score as follows: high/moderate disease activity (H-Mo/RA) if DAS28-CRP was ≥ 3.2 or into sustained clinical and US remission (Rem/RA) if DAS28-CRP was ≤ 2.6 for at least the last two consecutive follow-up visits without any therapeutic changes and without power-doppler (PD) signal at the US examination, as previously described [17]. Moreover, each Rem/RA patient was followed in an outpatient clinical setting to assess the occurrence of disease flare which was defined as the loss of the remission status (DAS28-CRP > 2.6) causing the need for a change in therapy by the treating rheumatologist, including the use of systemic and/or local glucocorticoids within the 24 months of follow-up.

### Ultrasound-guided minimally invasive synovial tissue biopsy

Each enrolled RA patient underwent a joint ultrasound (US) examination for assessment of synovitis according to the OMERACT definition and EULAR-OMERACT scoring system [22, 23] at baseline and at each follow-up visit. In particular, synovial membrane hypertrophy (SMH) and power Doppler (PD) grade were collected. Moreover, minimally invasive US-guided biopsy of the knee synovial tissue (ST) was performed at baseline for each RA patient, to evaluate the degree of synovitis using the hematoxylin and eosin (H&E) based Krenn synovitis score (KSS), as previously described [24, 25]. All ST specimens (at least 6–8 fragments) were stained for H&E and were examined by a trained pathologist unaware of any clinical and immunological patients’ characteristics [25]. KSS was used to define the histological synovitis grade as follows: 0–1, no synovitis; 2–4, low-grade synovitis; and KSS ≥ 5, moderate-high grade synovitis respectively.

### Assessment of health and disease status in enrolled patients

For each enrolled patient (both RA and FIBRO groups), health and disease status was evaluated by the Health Assessment Questionnaire (HAQ), the Visual Analog Scales for pain (VAS-Pain; 0–100 range with 0, absence of pain, to 100, worst pain ever; VAS-Pain more than 20 identifies an unacceptable pain) [26], the Functional Assessment of Chronic Illness Therapy-Fatigue scale (in this paper shorted as FACIT; measures the level of fatigue in various fields with 0–52 range: the higher the score, the less severe the symptoms of fatigue) [27], the Central Sensitization Inventory (CSI, a self-report test designed to identify patients who have symptoms that may be related to central sensitization or central sensitivity syndromes; a score of more than 40 indicates the presence of central sensitization process) [28], the General Health Questionnaire (GHQ, a self-report test designed to identify patients with psychiatric diseases; a score of at least 24 indicates the presence of mental disorders) [29] and the Rheumatoid Arthritis Impact of Disease (RAID) questionnaire (a RA validated patient-derived tool which assessed pain, functional disability, fatigue, sleep, coping, physical and emotional well-being; at an individual patient level, a score < 2 is deemed a patient-acceptable symptom-state (PASS)) [30, 31].

### Statistical analysis

Data were expressed as median and interquartile range (IQR) or percentage, as appropriate. Differences between continuous variables were evaluated using the Mann–Whitney test or the ANOVA test, as appropriate, while for categorical data, differences were calculated using the chi-square probability test. Correlations were performed using the Spearman rank test. Kaplan–Meier survival curve was evaluated using the Log-rank (Mantel-Cox) test to compare the flare occurrence between RA subgroup patients. A *p*-value ≤ 0.05 was considered statistically significant. Statistical analysis was performed using GraphPad (GraphPad Software, San Diego California USA, version 10.0.1) and SPSS Statistics (v. 26.0).

## Results

### Clinical and demographic characteristics associated with residual pain across RA disease

Table 1 summarizes the demographic and clinical features of the enrolled cohorts. In particular, the three sub-groups of patients differed in terms of age (ANOVA test, *p* = 0.02), but not for sex (*p* = 0.56), BMI (*p* = 0.66), and smoking habits (*p* = 0.06). Considering Rem/RA vs H-Mo/RA groups, no differences were found in terms of age (*p* = 0.29), sex (*p* = 0.57), and BMI (*p* = 0.69). Conversely, Rem/RA differed from H-Mo/RA in terms of seropositivity (65% vs 50%, respectively; *p* = 0.007), erosion status (24% vs 56%, respectively; *p* = 0.039), and DAS28-CRP (*p* < 0.0001) (Table 1).

**Table 1:** Demographic, clinical, immunological, ultrasonographic and histological characteristics of enrolled patients with RA and fibromyalgia

	Rem/RA (a) *N* = 68	H-Mo/RA (b) *N* = 29	FIBRO *N* = 30	*p* value*
Age (y)	57.5 (49.5–65.0)	59.0 (54.5–67.5)	47.0 (38.0–59.2)	ns
BMI (kg/mq)	24.0 (21.0–27.2)	25.5 (21.2–31.2)	26.0 (22.5–28.2)	ns
Female sex (*n*.; %)	57 (84)	20 (80)	27 (90)	ns
Smoking status (*n.*; %)	11 (16)	13 (45)	10 (38)	ns
Disease duration (y)	6.0 (3.2–13.7)	4.0 (0.5–12.2)		ns
RF/ACPA positivity	44 (65)	14 (50)		0.007
ESR (mm/1st h)	12.0 (6.5–27.5)	36.0 (15.5–73.5)		0.001
CRP (mg/l)	1.06 (0.50–2.15)	11.60 (2.45–37.70)		< 0.0001
DAS28-CRP	1.85 (1.39–2.11)	5.21 (4.23–5.62)		< 0.0001
CDAI	2.39 (1.10–4.37)	32.38 (19.76–35.41)		< 0.0001
SMH (cm)	0.82 (0.71–0.99)	1.01 (0.87–1.20)		0.002
PD grade	0	1 (1–2)		< 0.0001
KSS	2 (1–2)	5 (3–6)		< 0.0001
Steroids treatment (*n.*; %)	0	7 (24)		< 0.0001
MTX treatment (*n.*; %)	55 (81)	12 (40)		< 0.0001
bDMARDs treatment (*n.*; %)	55 (81)	7 (24)		< 0.0001

*p*-value ≤ 0.05 was considered statistically significant

*y*, years; *BMI*, Body Mass Index; *kg/ms*, kilogram per meter squared; *RF*, rheumatoid factor; *ACPA*, anti-citrullinated protein antibody; *ESR*, erythrocyte sedimentation rate; month; *mm/1ST h*, millimeter in the first hour; *CRP*, C-reactive protein; *mg/l*, milligram per liter; *DAS28-CRP*, CRP-based disease activity score of 28 joints; *CDAI*, clinical disease activity index; *SMH*, synovial membrane hypertrophy; *PD*, power Doppler; *KSS*, Krenn synovitis score; *MTX*, methotrexate; *bDMARDs*, biological Disease Modifying AntiRheumatic Drugs; *Rem/RA*, rheumatoid arthritis (RA) group of patient in remission; *H-Mo/RA*, high-moderate disease activity group; *FIBRO*, fibromyalgia group. Median and IQR range or frequency and percentages are shown as appropriated

*p value: Mann–Whitney test or chi square test as appropriated between Rem/RA and H-Mo/RA

Considering ultrasound features and synovitis degree, Rem/RA patients showed lower synovial membrane hypertrophy (SMH) (0.82 (0.71–0.99) cm) and KSS (2 (1–2)) compared to H-Mo/RA (PD grade: 1 (1–2), *p* < 0.0001; SMH: 1.01 (0.87–1.20) cm, *p* = 0.002; KSS: 5 (3–6), *p* < 0.0001) (Supplementary Fig. 1a). Comparing the two RA sub-groups, 34 (50%) Rem/RA patients presented no histological signs of synovitis compared to 2 (9%, *p* = 0.0004) H-Mo/RA patients; moreover, 34 (50%) Rem/RA patients presented a low grade of synovitis than 8 (35%, *p* < 0.0001) H-Mo/RA patients; none of Rem/RA patients presented a moderate-high grade of synovitis compared to 13 (56%, *p* < 0.0001) H-Mo/RA patients (Supplementary Fig. 1b).

Considering concomitant pharmacological treatments, none of Rem/RA patients was using steroids compared to 7 (24%) of H-Mo/RA (*p* < 0.0001); Rem/RA differed also from H-Mo/RA for methotrexate (MTX) use (81% vs 40% respectively; *p* < 0.0001) and bDMARDs treatment (81% vs 24% respectively; *p* < 0.0001) (Table 1). These differences were due to the heterogeneous disease stage of H-Mo/RA patients, including early RA, MTX-not responder, and bDMARDs-not responder RA. Furthermore, within the b-DMARDs-induced Rem/RA sub-group, 36 (65%) RA patients were treated with TNFi, 12 (22%) with anti-IL6R, and 7 (13%) with anti-CTLA4-Ig respectively. None of the Rem/RA patients was in drug-free remission.

To investigate the potential role of treatment regimens on RP in RA, Rem/RA patients were stratified based on a combination regimen (csDMARDs-only vs csDMARDs + bDMARDs vs bDMARDs-only) (Suppl. Figure 2a) or on bDMARDs treatment (csDMARDs-only vs TNFi vs IL6i vs CTLA4) (Suppl. Figure 2b). In particular, VAS-Pain was not affected by combination regimen or by bDMARDs treatment in Rem/RA patients.

### Rem/RA patients still reported unacceptable residual pain despite sustained clinical and US remission and regardless of the degree of histological synovitis

**Fig. 1:**
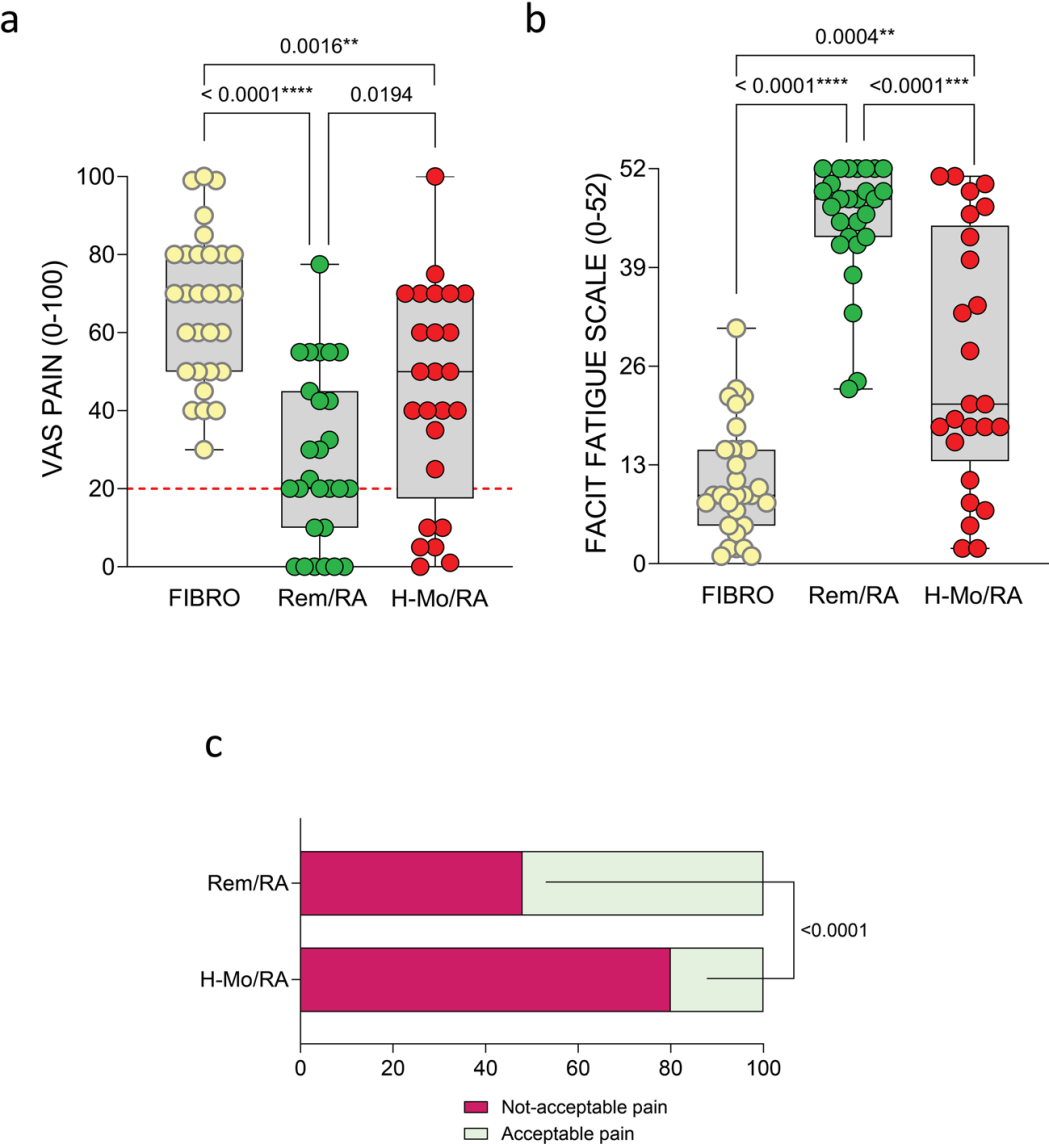
Pain assessment in RA patients across disease stages and fibromyalgia patients. **a**, **b** VAS-Pain and FACIT scores in RA patients stratified by disease stage namely DAS28-CRP based remission (Rem/RA) and DAS28-CRP based high-moderate disease activity (H-Mo/RA) and fibromyalgia patients (FIBRO). ANOVA test and Mann–Whitney test were used. Every dot is a patient. The median is shown as a blank line in the box, interquartile range is delimited by the box. *p*-value ≤ 0.05 was considered statistically significant. The dotted red line indicates VAS-Pain > 20 as threshold unacceptable pain; **c** rate of unacceptable pain in Rem/RA vs H-Mo/RA patients in the enrolled cohort. The chi-square test was used. *p*-value ≤ 0.05 was considered statistically significant

As shown in Fig. 1a, b, Rem/RA patients presented lower grade of pain (VAS-Pain 20.0 (10.0–45.0)) and fatigue (FACIT 46.0 (33.0–50.0)) compared to H-Mo/RA and FIBRO groups, who presented higher grade of VAS-Pain (H-Mo/RA 50.0 (17.5–70.0), *p* = 0.0194; FIBRO 70.0 (50.0–80.0), *p* < 0.0001) and lower score of FACIT (H-Mo/RA 21.0 (13.5–44.5), *p* < 0.0001; FIBRO 9.0 (5.0–15.0), *p* < 0.0001).

Furthermore, despite the sustained clinical and ultrasound remission achievement, 33 (48%) Rem/RA patients still complained of unacceptable pain (VAS-Pain > 20) compared to 23 (80%) H-Mo/RA patients (*p* < 0.0001) (Fig. 1c).

Considering only Rem/RA, residual disease activity measured by DAS28-CRP correlated with VAS-Pain (*r* 0.433, *p* < 0.0001) and FACIT (*r* − 0.417, *p* < 0.0001), but not with KSS (*p* = 0.336 for VAS-Pain and *p* = 0.337 for FACIT, respectively). In particular, when comparing Rem/RA patients with no histological synovitis (KSS < 2) vs histological synovitis presence (KSS ≥ 2), they presented comparable levels of VAS-Pain (*p* = 0.314), FACIT (*p* = 0.300), RAID (*p* = 0.617), CSI (*p* = 0.320), and GHQ (*p* = 0.105). Based on these, the whole Rem/RA group was considered in further analysis regardless of synovitis degree.

### A small rate of RA patients showed residual pain as part of a central sensitivity syndrome or psychiatric disorders despite sustained remission achievement

To investigate the influence of the nociplastic pain in Rem/RA patients, the presence of central sensitization using the CSI questionnaire and psychiatric disorders by GHQ was evaluated. In particular, Rem/RA patients showed a lower CSI score (16.0 (6.0–31.0)), compared to H-Mo/RA (29.0 (17.5–49.0); *p* = 0.0007) and FIBRO (59.0 (49.7–67.2); *p* < 0.0001) (Suppl. Figure 3a). Considering the CSI cutoff of 40, which indicates the presence of central sensitization of pain, only 7 (10%) Rem/RA patients had central pain sensitization, comparing to 8 H-Mo/RA (26%; *p* = 0.0032) and 30 FIBRO patients (100%;* p* < 0.0001) (Suppl. Figure 3b). Similarly, Rem/RA showed the lowest degree of GHQ score (16.0 (10.0–25.0)) when compared to H-Mo/RA (24.0 (13.0–37.0); *p* = 0.028) and FIBRO (37.5 (23.7–48.2); *p* < 0.0001) (Suppl. Figure 3c). Considering the GHQ cutoff of 24 which indicates the presence of psychiatric disorder, only 18 (26%) Rem/RA patients had a result compatible with concomitant psychiatric disorder, compared to 15 (52%; *p* = 0.020) of H-Mo/RA and 23 (77%; *p* < 0.0001) of FIBRO patients (Suppl. Figure 3d), respectively.

### Residual pain and fatigue as part of the patient dissatisfaction with the disease management, despite sustained clinical and US remission

**Fig. 2:**
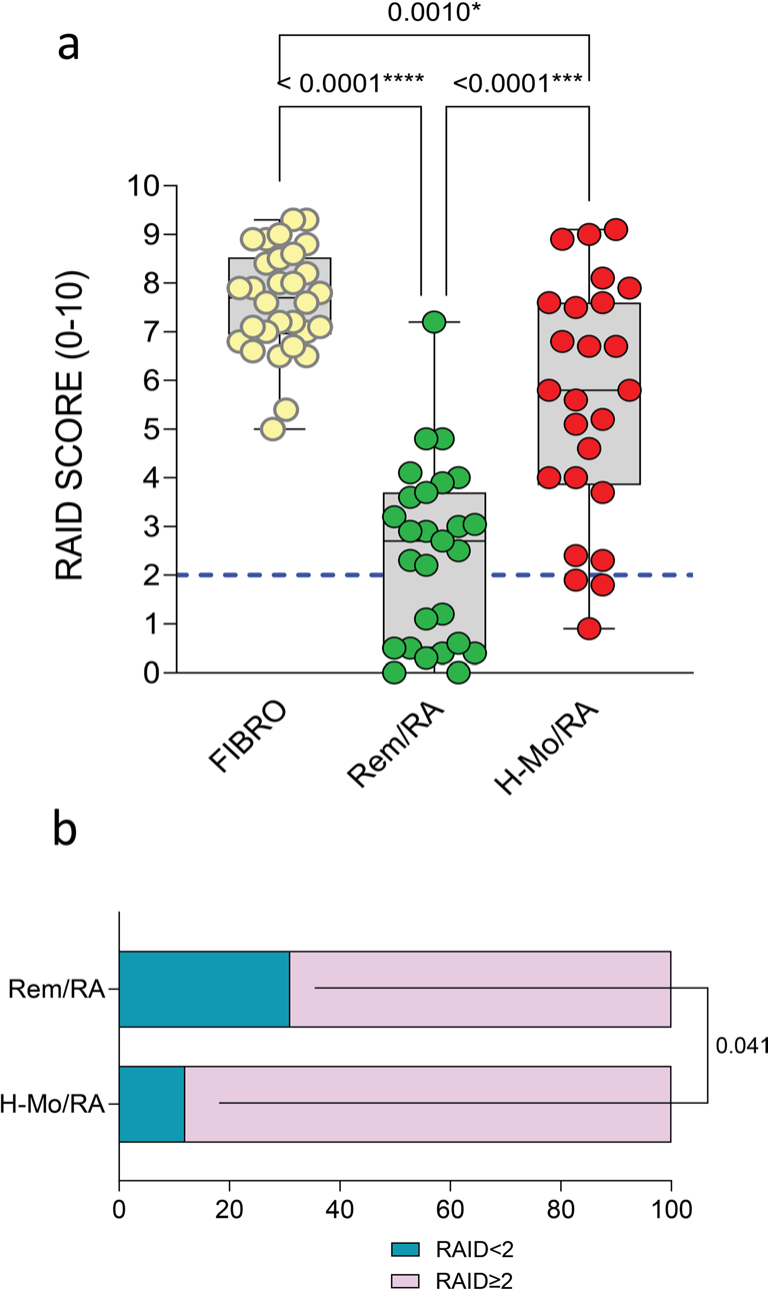
RAID score in RA in different clinical stages. **a** RAID score in Rem/RA, H-Mo/RA, and FIBRO groups. ANOVA test and Mann–Whitney test were used. Every dot is a patient. The median is shown as a blank line in the box, interquartile range is delimited by the box. The blue dotted line indicates the RAID cutoff of 2. *p*-value ≤ 0.05 was considered statistically significant. **b** Rate of patient-acceptable symptom state (RAID cutoff = 2) in Rem/RA and H-Mo/RA. The chi-square test was used. *p*-value ≤ 0.05 was considered statistically significant. FIBRO, fibromyalgia patients; Rem/RA, rheumatoid arthritis patients in DAS28-CRP-based remission; H-Mo/RA, rheumatoid arthritis patients in DAS28-CRP based high/moderate disease activity

As suggested by the results obtained so far, it can be excluded that the residual pain is driven by the inflammatory process in Rem/RA patients. Furthermore, it was found that non-inflammatory mechanisms explained to a small extent the presence of residual pain. Therefore, it was hypothesized that pain and fatigue were part of the patient’s dissatisfaction with the general state of health. To prove this hypothesis, a RAID questionnaire was administered to RA patients. As shown in Fig. 2a, Rem/RA patients reached the lowest score (3.12 (1.00–5.30)), compared to H-Mo/RA (5.80 (3.85–7.60); *p* = 0.0003) and FIBRO (7.70 (6.95–8.52) patients; *p* < 0.0001). When considering the RAID cutoff of 2 for PASS, only 21 (31%) Rem/RA patients were in PASS compared to 3 (12%; *p* = 0.041) H-Mo/RA and 0 (0%; *p* = 0.0003) FIBRO patients (Fig. 2b).

Furthermore, stratifying the Rem/RA group based on PASS achievement (RAID score < 2 as SAT-Rem/RA and RAID ≥ 2 as UNSAT-Rem/RA respectively), the two groups did not differ in terms of age (*p* = 0.18), BMI (*p* = 0.47), smoking habits (*p* = 0.70), MTX use (*p* = 0.19), csDMARDs (*p* = 0.217), and bDMARDs treatment (*p* = 0.357).

**Fig. 3:**
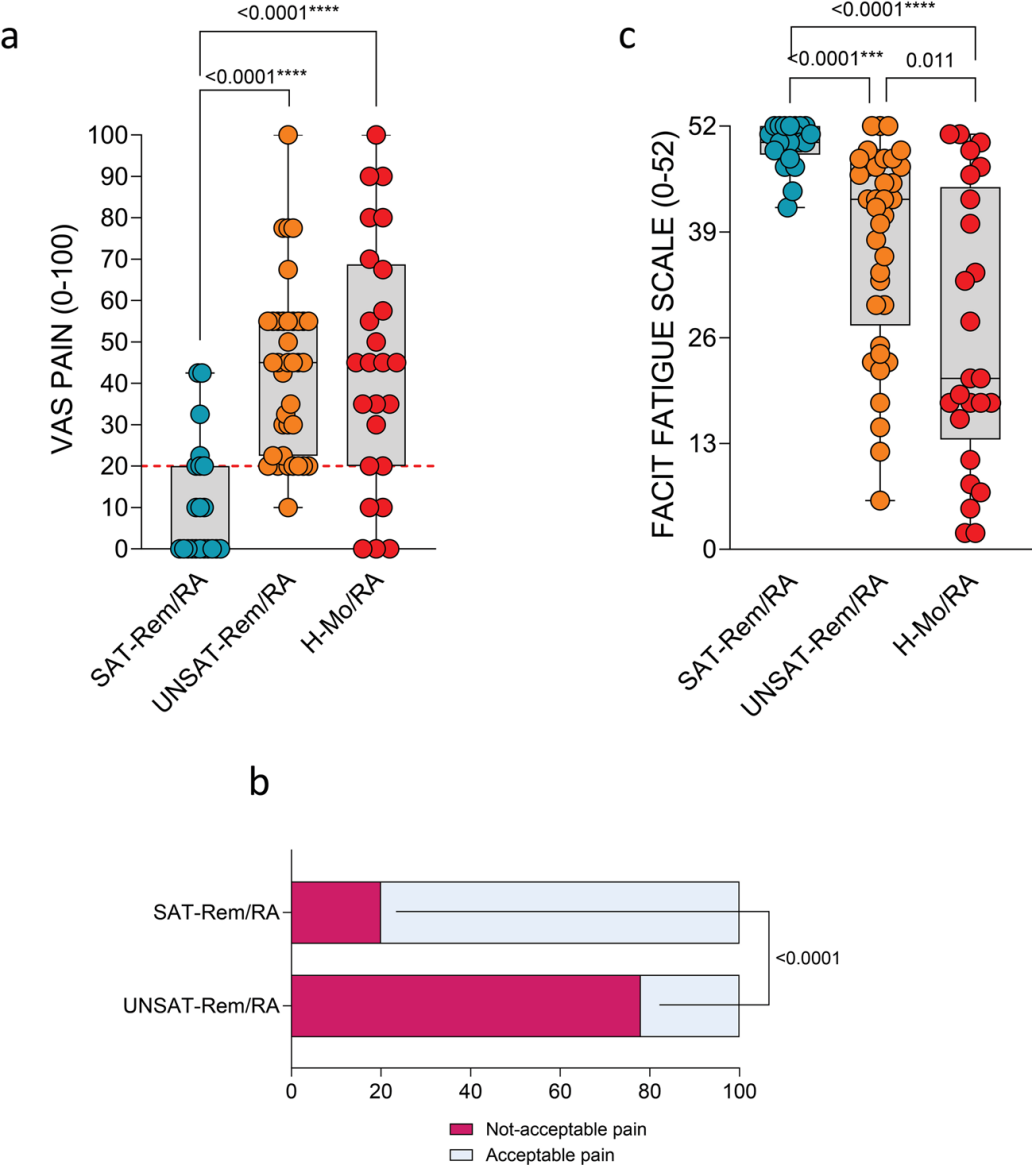
Residual pain and rates of patient dissatisfaction in RA in remission.** a** VAS-Pain score in Rem/RA with RAID < 2 (SAT), Rem/RA with RAID ≥ 2 (UNSAT), or H-Mo/RA groups. ANOVA test and Mann–Whitney test were used. Every dot is a patient. The median is shown as a blank line in the box, interquartile range is delimited by the box. The dotted orange line indicates the VAS-Pain cutoff of 20. **b** Rate of unacceptable pain in Rem/RA based on their RAID score (RAID < 2 (SAT), Rem/RA with RAID ≥ 2(UNSAT)). **c** FACIT score in Rem/RA with RAID < 2 (SAT), Rem/RA with RAID ≥ 2 (UNSAT), or H-Mo/RA groups. ANOVA test and Mann–Whitney test were used. Every dot is a patient. The median is shown as a blank line in the box, interquartile range is delimited by the box. *p*-value ≤ 0.05 was considered statistically significant. Rem/RA, rheumatoid arthritis patients in DAS28-CRP-based remission; H-Mo/RA, rheumatoid arthritis patients in DAS28-CRP-based high/moderate disease activity

Conversely, the SAT-Rem/RA group was less likely female (71%) than UNSAT-Rem/RA (92%, *p* = 0.039). Moreover, SAT-Rem/RA group presented a lower grade of pain (VAS-Pain: 0 (0–20.0)) (Fig. 3a), a lower percentage of patients with an unacceptable pain (19%) (Fig. 3b) and a lower grade of fatigue (FACIT: 50.0 (48.5–52.0)) (Fig. 3c) compared to the UNSAT-Rem/RA patients (VAS-Pain 50.0 (30.0–67.5), *p* < 0.0001; percentage of patients with an unacceptable pain: 38 (81%), *p* < 0.0001; FACIT 38.0 (18.0–47.0), *p* < 0.0001, respectively). Interestingly, VAS-Pain and fatigue were comparable between UNSAT-Rem/RA and H-Mo/RA (50.0 (30.0–67.5) *vs* 50.0 (17.5–70.0), *p* > 0.05; 38.0 (18.0–47.0) vs 21.0 (13.5–44.5), *p* > 0.05, respectively).

Finally, Rem/RA patients were followed for a period of 24 months and 9 patients showed a flare of disease with a median time from remission achievement of 6 months. Considering RAID stratification, the percentage of SAT-Rem/RA patients who presented a disease flare did not differ from UNSAT-Rem/RA over the 24 months of follow-up (55% vs 45% respectively, *p* = 0.4916) (Suppl. Figure 4).

### Female rheumatoid arthritis patients reported more likely residual pain than male patients even in remission status

**Fig. 4:**
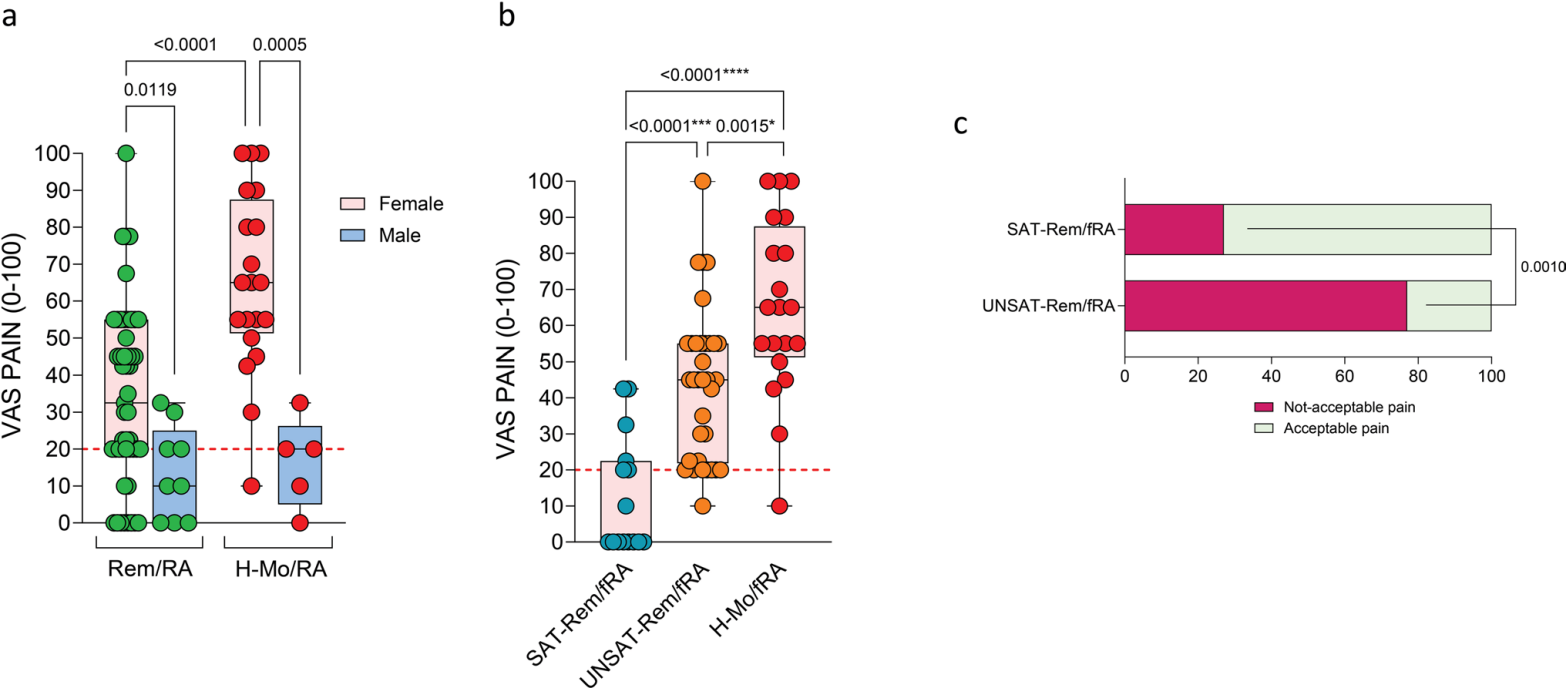
Residual pain is associated with the female sex in a remission state. **a** VAS-Pain score in Rem/RA and H-Mo/RA stratified based on sex. Mann–Whitney test was used. Every dot is a patient. The median is shown as a blank line in the box; the interquartile range is delimited by the box. The orange dotted line indicates the VAS-Pain cutoff = 20.** b** VAS-Pain score in female Rem/RA (Rem/fRA) stratified based on RAID < 2 (SAT), Rem/RA with RAID ≥ 2 (UNSAT), and female H-Mo/RA (H-Mo/fRA) groups. ANOVA and Mann–Whitney test were used. Every dot is a patient. The median is shown as a blank line in the box; the interquartile range is delimited by the box. The orange dotted line indicates the VAS-Pain cutoff = 20.** c** Rate of unacceptable pain in female Rem/RA (Rem/fRA) based on their RAID score (RAID < 2 (SAT) and Rem/RA with RAID ≥ 2 (UNSAT) respectively). The chi-square test was used. Rem/RA, rheumatoid arthritis patients in DAS28-CRP-based remission; H-Mo/RA, rheumatoid arthritis patients in DAS28-CRP-based high/moderate disease activity

To investigate the potential role of sex in the perception of RP, female and male RA patients were compared within Rem/RA and H-Mo/RA groups (Table 2). In particular, despite a comparable level of disease burden in terms of DAS28-CRP, PD grade, and KSS between female and male RA patients within each subgroup, female Rem/RA patients reported higher VAS-Pain (32.5 (20.0–55.0)) compared to male Rem/RA (10.0 (0–25.0); *p* = 0.0119). Similarly, in the H-Mo/RA group, female patients reported higher VAS-Pain (65.0 (51.2–87.5)) compared to male patients (20.0 (5.0–26.2); *p* = 0.0005) (Fig. 4a). Conversely, male RA patients reported comparable VAS-pain among remission (10.0 (0–25.0)) and active stage (20.0 (5.0–26.2); *p* > 0.05), despite significantly different synovitis degree (1 (1–3) in remission male RA vs 6 (3–6) in active stage RA patients, *p* > 0.05). Considering only female RA patients and stratifying them based on RAID PASS achievement, PASS female Rem/RA (SAT-Rem/fRA) patients presented lower VAS-Pain (0 (0–22.5)) compared to not-PASS female Rem/RA (UNSAT-REM/fRA) (45.0 (21.9–55.0); *p* < 0.0001) and H-Mo/fRA (65.0 (51.2–87.5); *p* < 0.0001) (Fig. 4b).

**Table 2:** Demographic, clinical, immunological, ultrasonographic, and histological characteristics of female and male RA patients

	Female Rem/RA (a) *N* = 57	Male Rem/RA (b) *N* = 11	Female H-Mo/RA (c) *N* = 23	Male H-Mo/RA (d) *N* = 6	*p* value (a vs b)	*p* value (c vs d)
Age (y)	59.0 (50.0–64.5)	54.0 (45.5–68.0)	60.0 (49.5–67.7)	57.0 (55.5–70.0)	ns	ns
BMI (kg/mq)	24.0 (21.0–28.0)	26.0 (25.0–27.5)	25.0 (21.2–31.2)	26.0 (19.2–32.0)	ns	ns
Smoking status (*n.*; %)	9 (16)	2 (25)	8 (35)	5 (80)	ns	0.029
Disease duration (y)	6.0 (3.0–13.0)	6.0 (4.5–14.0)	4.0 (0.5–16.0)	2.0 (0.2–7.0)	ns	ns
RF/ACPA positivity	36 (63)	8 (72)	12 (50)	2 (40)	ns	ns
ESR (mm/1st h)	16.0 (8.5–31.2)	7.0 (3.5–14.5)	39.0 (16.0–78.7)	31.0 (13.0–45.0)	0.021	ns
CRP (mg/l)	1.02 (0.50–2.40)	0.50 (0.20–1.95)	12.70 (3.12–43.20)	8.50 (1.20–12.70)	ns	ns
DAS28-CRP	1.85 (1.37–2.12)	1.90 (1.67–2.08)	5.22 (4.63–5.72)	4.14 (3.44–4.87)	ns	ns
CDAI	2.56 (1.10–4.52)	2.15 (1.20–4.60)	33.13 (22.78–39.03)	19.66 (15.97–26.69)	ns	0.027
SMH (cm)	0.82 (0.68–0.99)	0.91 (0.75–1.07)	1.07 (0.94–1.22)	0.74 (0.64–1.05)	ns	0.031
PD grade	0	0	1.5 (1.0–2.0)	1 (0–1.5)	ns	ns
KSS	2 (1–2)	1 (1–3)	5 (3–6)	6 (3–6)	ns	ns
Steroids treatment (*n.*; %)	0	0	5 (20)	2 (40)	ns	ns
MTX treatment (*n.*; %)	46 (81)	9 (82)	10 (43)	2 (40)	ns	ns
bDMARDs treatment (*n.*; %)	46 (81)	9 (82)	6 (25)	1 (20)	ns	ns

*p*-value ≤ 0.05 was considered statistically significant

*y*, years; *BMI*, Body Mass Index; *kg/ms*, kilogram per meter squared; *RF*, rheumatoid factor; *ACPA*, anti-citrullinated protein antibody; *ESR*, erythrocyte sedimentation rate; month; *mm/1ST h*, millimeter in the first hour; *CRP*, C-reactive protein; *mg/l*, milligram per liter; *DAS28-CRP*, CRP-based disease activity score of 28 joints; *CDAI*, clinical disease activity index; *SMH*, synovial membrane hypertrophy; *PD*, power Doppler; *KSS*, Krenn synovitis score; *MTX*, methotrexate; *bDMARDs*, biological Disease Modifying AntiRheumatic Drugs; *Rem/RA*, rheumatoid arthritis (RA) group of patient in remission; *H-Mo/RA*, high-moderate disease activity group. Median and IQR range or frequency and percentages are shown as appropriate

*p value: Mann–Whitney test or chi square test as appropriated between female and male RA patients within each subgroups

Finally, 11 (73%) SAT-Rem/fRA patients presented an acceptable pain compared to 8 (23%) UNSAT-Rem/fRA patients (*p* = 0.001) (Fig. 4c).

## Discussion

When RA is not controlled by adequate therapy, the disease is associated with joint swelling associated with pain. Conversely, it is expected that the achievement of the state of remission would lead to a complete regression of the pain symptom, but this, unfortunately, does not always occur [12–14]. Despite multiple novel treatments against RA, RP remains an urgent medical need in the management of RA [32].

In our study, we described how in a cohort of RA patients in sustained clinical and ultrasound remission a portion of the patients still complained of RP. In particular, this cross-sectional study of Italian RA patients showed that, despite intensive treatment and low disease activity scores, 48% of Rem/RA patients reported the presence of unacceptable pain, regardless of the degree of synovitis, which was at most mild (KSS ≤ 4) in half of the Rem/RA patients. Likewise, also fatigue was still reported by a part of the Rem/RA group. Excluding the inflammatory cause of RP, we investigated the nociplastic nature of the RP through the administration of two questionnaires (CSI and GH questionnaire), and we found that in the Rem/RA group, the psychiatric and central sensitization disorders could explain only minimally part of the persistence of the RP (26% and 10% respectively).

Based on those data, we wondered if pain and fatigue were used by the RA patients to communicate the state of discomfort and non-acceptance of their own disease regardless of achieving clinician-defined remission status. To test this hypothesis, patients answered the RAID questionnaire, whose validated score defines the patient-acceptable symptom state (PASS) [30, 31].

We found that up to 69% of RA patients in clinician-defined remission status did not actually feel in remission. Stratifying the Rem/RA group on the basis of the achievement of the PASS status, the patients satisfied with their own state of health (SAT-Rem/RA) presented less residual pain and fatigue compared to the unsatisfied ones (UNSAT-Rem/RA), who were characterized by a VAS-Pain and fatigue comparable to H-Mo/RA group. Furthermore, 78% of UNSAT-Rem/RA patients still complained of unacceptable pain despite achieving sustained clinical and ultrasound remission status. Moreover, the two SAT-Rem/RA and UNSAT-Rem/RA groups did not differ in terms of disease flare up to 24 months after the enrolment in the study, suggesting the absence of residual nociceptive pain, also supported by the absence of differences in residual synovitis as evidenced by the KSS analyses.

Finally, female RA patients complained of more pain than male RA patients independently of disease status, as already described in RA by other authors [33, 34]. In particular, female RA patients in clinically defined remission but who were not satisfied with their health status perceived a higher degree of pain compared to female RA who were satisfied with their health status.

Pain is the most important symptom that prompts the patient affected by RA to consult the rheumatologist [35]. Likewise, pain is also a self-tool which RA patients use to measure the effectiveness of the treatment [36]. However, the perception of pain can be influenced by numerous aspects such as biological, psychological, and social factors [5, 37], so it cannot be used as an objective measure of the therapy’s effectiveness. Conversely, the rheumatologist should not neglect RP in RA because there is a risk of compromising the patient’s therapeutic adherence [38] and the patient’s trust in the clinician [39], as well as, above all, the patient’s quality of life with the risk of chronic pain. On the other hand, RP should not be rapidly dismissed as nociplastic pain, associated with the presence of fibromyalgia, even when the evidence does not indicate the presence of a central sensitization or psychiatric disorders, as described in our cohort. As hypothesized, in fact, pain (as well as fatigue) can represent a means by which the patients communicate their state of non-acceptance of the disease and/or dissatisfaction, probably due to the fact of having a chronic pathology and living without a clear time of suspension of therapies [40]. Furthermore, there is growing evidence that there are biological mechanisms that directly regulate and amplify pain in RA, independently of inflammation. Recently, Bai Z. et al. showed that synovial lining fibroblasts express pain-related genes that enhance the growth of pain-sensing neurons in regions of RA synovial hypertrophy (i.e., netrin-4, within the GbGMI-identified pain-associated gene module) [41].

Based on these issues, it is crucial to understand the underlying nature of RP in RA patients in order to provide the most appropriate pharmacological and non-pharmacological approaches. In particular, for the former, as described by our results, csDMARDs or bDMARDs treatment did not influence RP perception when the pain was not due to nociceptive or nociplastic mechanisms. This insight is particularly relevant in clinical settings when arthritis appears to be clinically, ultrasonographically, and histologically well-controlled yet the patient continues to report persistent pain. Such scenarios can lead to inappropriate therapy changes that fail to address the root cause of pain issues or lead to adding other useless drugs, potentially reducing patient adherence and compliance to the therapies [42], simultaneously contributing to healthcare costs increasing for both the system and society [43].

On the other hand, for the latter approach, acceptance and commitment therapy (ACT) and cognitive behavioral therapy (CBT) are indicated in the treatment of chronic pain conditions such as fibromyalgia [5, 44–46]. Notably, Marques AP and Antunes MD’s group described various tools to support self-care and education, including a mobile application [47], an e-book [8], and an educational program aimed at promoting health in fibromyalgia [48]. These tools have consistently demonstrated significant benefits in terms of reducing pain, disability, depression, anxiety, and fatigue in fibromyalgia patients.

Based on these findings, we can speculate on the potential role of ACT, CBT, and education programs in RA management, regardless of the disease activity. These approaches may help patients accept their health status and illness, potentially alleviating the perception of residual pain and reducing its impact on their quality of life.

The primary limitation of this study could be its monocentric design. Given that pain perception and expression are significantly influenced by societal and cultural aspects, it would be valuable to evaluate RP in RA across different settings. To enhance the validity and generalizability of future findings, the use of validated questionnaires, the adoption of a stringent definition of clinical remission (taking into account clinical, ultrasound, and histological features), and the exclusion of RA patients with comorbid fibromyalgia are recommended.

In conclusion, a multidisciplinary approach to the treatment of pain in patients affected by RA is needed both in the active stages of the disease and when remission status is achieved. It can be useful to treat pain with the appropriate pharmacological and non-pharmacological treatments to educate RA patients to accept their own disease due to the fact that the acceptable patient health and disease status can affect the pain and fatigue perception. Furthermore, it can be useful to take into account also the sex differences in the perception of pain between male and female RA patients to better manage residual pain.

## Supplementary Information

Below is the link to the electronic supplementary material.

Supplementary Figure 1. a: Krenn Synovitis Score in RA patients stratified by disease stage namely DAS28-CRP based remission (Rem/RA) and DAS28-CRP based high-moderate disease activity (H-Mo/RA). Mann–Whitney test was used. Every dot is a patient. Median is shown as a blank line in the box, interquartile range is delimited by the box. P-value ≤ 0.05 was considered statistically significant. b: cumulative frequencies of Krenn Synovitis Score (KSS) within Rem/RA and H-Mo/RA groups. Rem/RA patients presented no synovitis (KSS: 0–1) or low-grade synovitis (KSS: 2–4) compared to H-Mo/RA characterized by high grade synovitis (KSS ≥ 5). P-value ≤ 0.05 was considered statistically significant. (JPG 2901 KB)

Supplementary Figure 2. a: treatment regimen (csDMARDs-only vs csDMARDs + bDMARDs vs bDMARDs-only) did not impact on VAS-Pain in Rem/RA patients. ANOVA test and Mann–Whitney test were used. Every dot is a patient. Median is shown as a blank line in the box, interquartile range is delimited by the box. P-value ≤ 0.05 was considered statistically significant. b: bDMARDs treatment (along csDMARDs-only treatment) did not impact on VAS-Pain in Rem/RA patients. ANOVA test and Mann–Whitney test were used. Every dot is a patient. Median is shown as a blank line in the box, interquartile range is delimited by the box. P-value ≤ 0.05 was considered statistically significant. csDMARDs: conventional synthetic DMARDs; bDMARDs: biological DMARDs. (JPG 3077 KB)

Supplementary Figure 3. a-b: Central Sensitization Inventory (CSI)scores and rate in RA patients stratified by disease stage namely DAS28-CRP based remission (Rem/RA) and DAS28-CRP based high-moderate disease activity (H-Mo/RA) and fibromyalgia patients (FIBRO). ANOVA test and Mann–Whitney test were used to compare CSI scores. Chi square test was used to compare percentages. Every dot is a patient. Median is shown as a blank line in the box, interquartile range is delimited by the box. P-value ≤ 0.05 was considered statistically significant. c-d: General Health Questionnaire (GHQ) scores and rate in RA patients stratified by disease stage namely DAS28-CRP based remission (Rem/RA) and DAS28-CRP based high-moderate disease activity (H-Mo/RA) and fibromyalgia patients (FIBRO). ANOVA test and Mann–Whitney test were used to compare GHQ scores. Chi square test was used to compare percentages. Every dot is a patient. Median is shown as a blank line in the box, interquartile range is delimited by the box. P-value ≤ 0.05 was considered statistically significant. (JPG 3498 KB)

Supplementary Figure 4. DAS28-CRP based remission (Rem/RA) patients survival curve stratified based on RAID score (RAID < 2 (SAT), RAID ≥ 2 (UNSAT)). Log-rank (Mantel-Cox) test to evaluate the difference in flare occurrence between RA subgroup patients. A p-value ≤ 0.05 was considered statistically significant. (JPG 1698 KB)
