# Supplementary figures and images for: Residual pain and fatigue are affected by disease perception in rheumatoid arthritis in sustained clinical and ultrasound remission
Source: Clin Rheumatol. 2025 Jan 22;44(3):1019–29. doi: 10.1007/s10067-025-07331-0 (PMC11865154; doi:10.1007/s10067-025-07331-0)

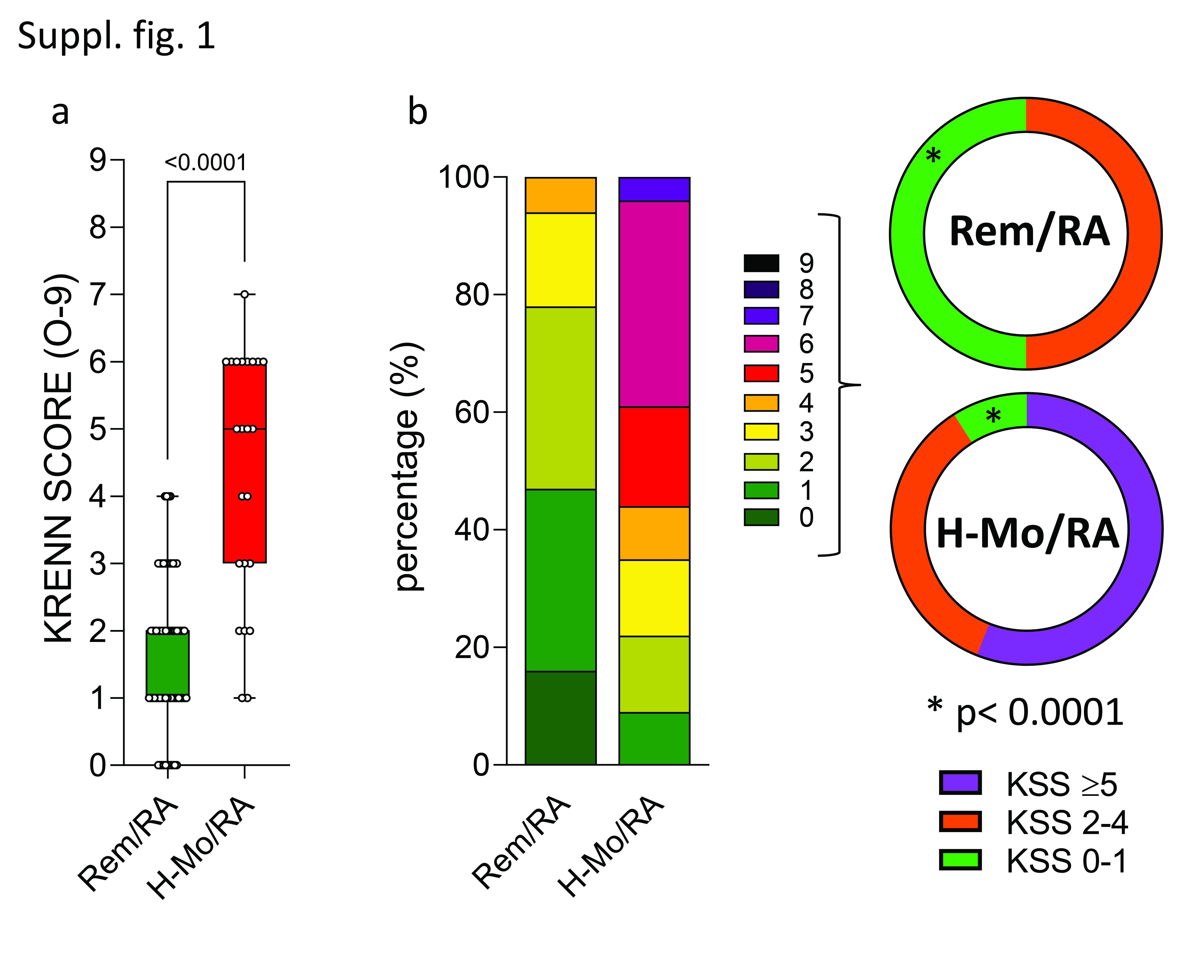

Supplement: Supplementary file 1 — Supplementary Figure 1. a: Krenn Synovitis Score in RA patients stratified by disease stage namely DAS28-CRP based remission (Rem/RA) and DAS28-CRP based high-moderate disease activity (H-Mo/RA). Mann–Whitney test was used. Every dot is a patient. Median is shown as a blank line in the box, interquartile range is delimited by the box. P-value ≤ 0.05 was considered statistically significant. b: cumulative frequencies of Krenn Synovitis Score (KSS) within Rem/RA and H-Mo/RA groups. Rem/RA patients presented no synovitis (KSS: 0–1) or low-grade synovitis (KSS: 2–4) compared to H-Mo/RA characterized by high grade synovitis (KSS ≥ 5). P-value ≤ 0.05 was considered statistically significant. (JPG 2901 KB) [file 10067_2025_7331_MOESM1_ESM.jpg]

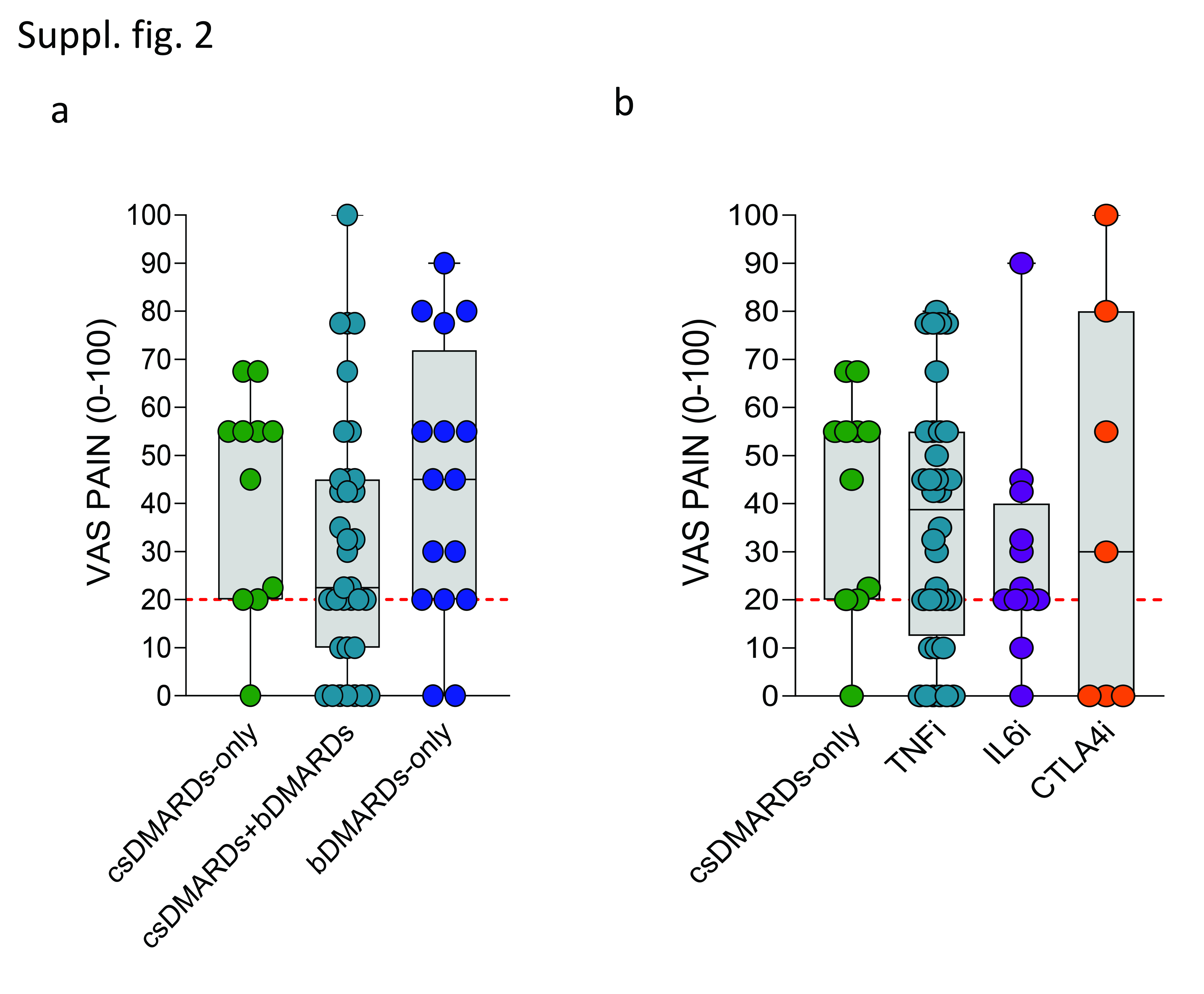

Supplement: Supplementary file 2 — Supplementary Figure 2. a: treatment regimen (csDMARDs-only vs csDMARDs + bDMARDs vs bDMARDs-only) did not impact on VAS-Pain in Rem/RA patients. ANOVA test and Mann–Whitney test were used. Every dot is a patient. Median is shown as a blank line in the box, interquartile range is delimited by the box. P-value ≤ 0.05 was considered statistically significant. b: bDMARDs treatment (along csDMARDs-only treatment) did not impact on VAS-Pain in Rem/RA patients. ANOVA test and Mann–Whitney test were used. Every dot is a patient. Median is shown as a blank line in the box, interquartile range is delimited by the box. P-value ≤ 0.05 was considered statistically significant. csDMARDs: conventional synthetic DMARDs; bDMARDs: biological DMARDs. (JPG 3077 KB) [file 10067_2025_7331_MOESM2_ESM.jpg]

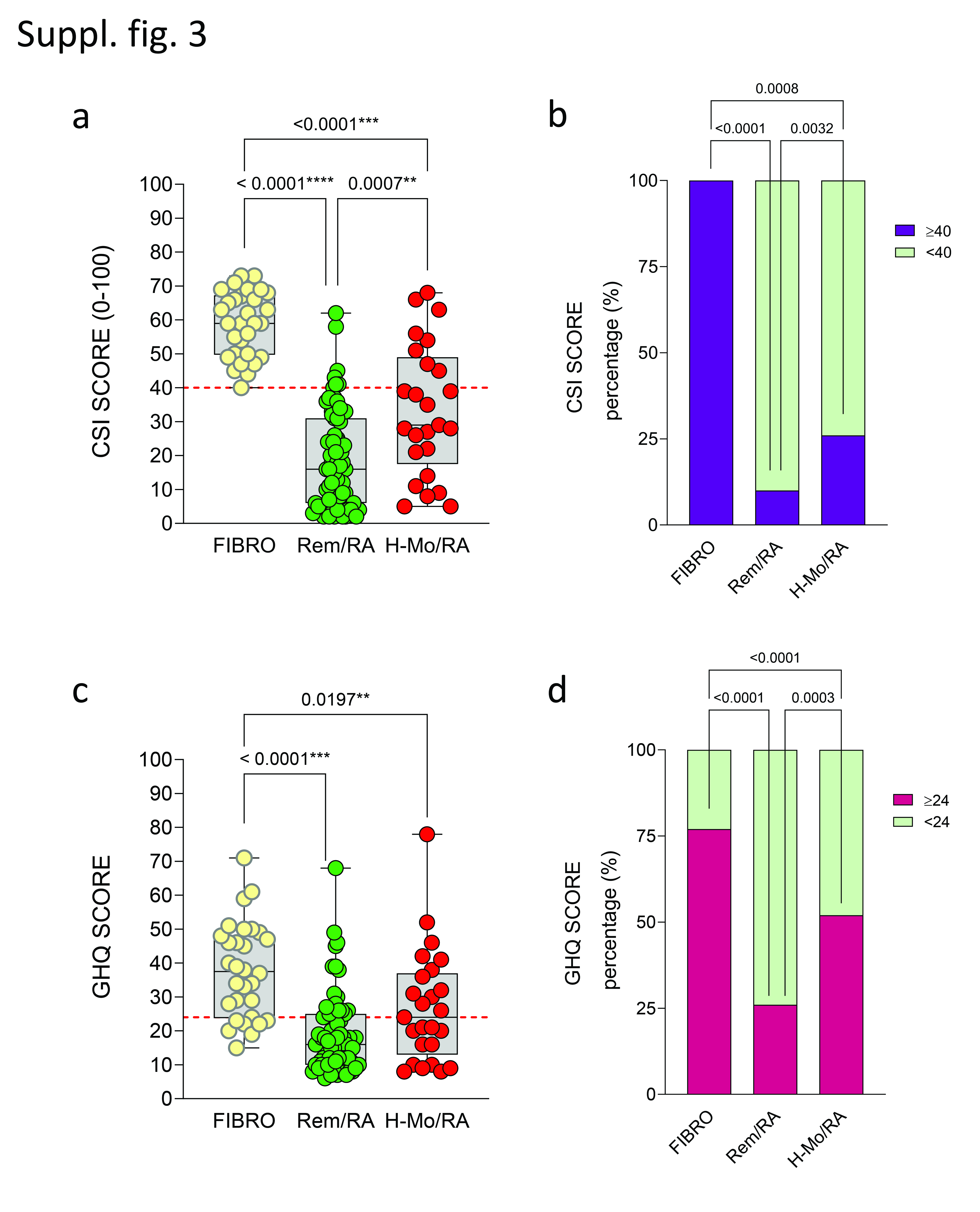

Supplement: Supplementary file 3 — Supplementary Figure 3. a-b: Central Sensitization Inventory (CSI)scores and rate in RA patients stratified by disease stage namely DAS28-CRP based remission (Rem/RA) and DAS28-CRP based high-moderate disease activity (H-Mo/RA) and fibromyalgia patients (FIBRO). ANOVA test and Mann–Whitney test were used to compare CSI scores. Chi square test was used to compare percentages. Every dot is a patient. Median is shown as a blank line in the box, interquartile range is delimited by the box. P-value ≤ 0.05 was considered statistically significant. c-d: General Health Questionnaire (GHQ) scores and rate in RA patients stratified by disease stage namely DAS28-CRP based remission (Rem/RA) and DAS28-CRP based high-moderate disease activity (H-Mo/RA) and fibromyalgia patients (FIBRO). ANOVA test and Mann–Whitney test were used to compare GHQ scores. Chi square test was used to compare percentages. Every dot is a patient. Median is shown as a blank line in the box, interquartile range is delimited by the box. P-value ≤ 0.05 was considered statistically significant. (JPG 3498 KB) [file 10067_2025_7331_MOESM3_ESM.jpg]

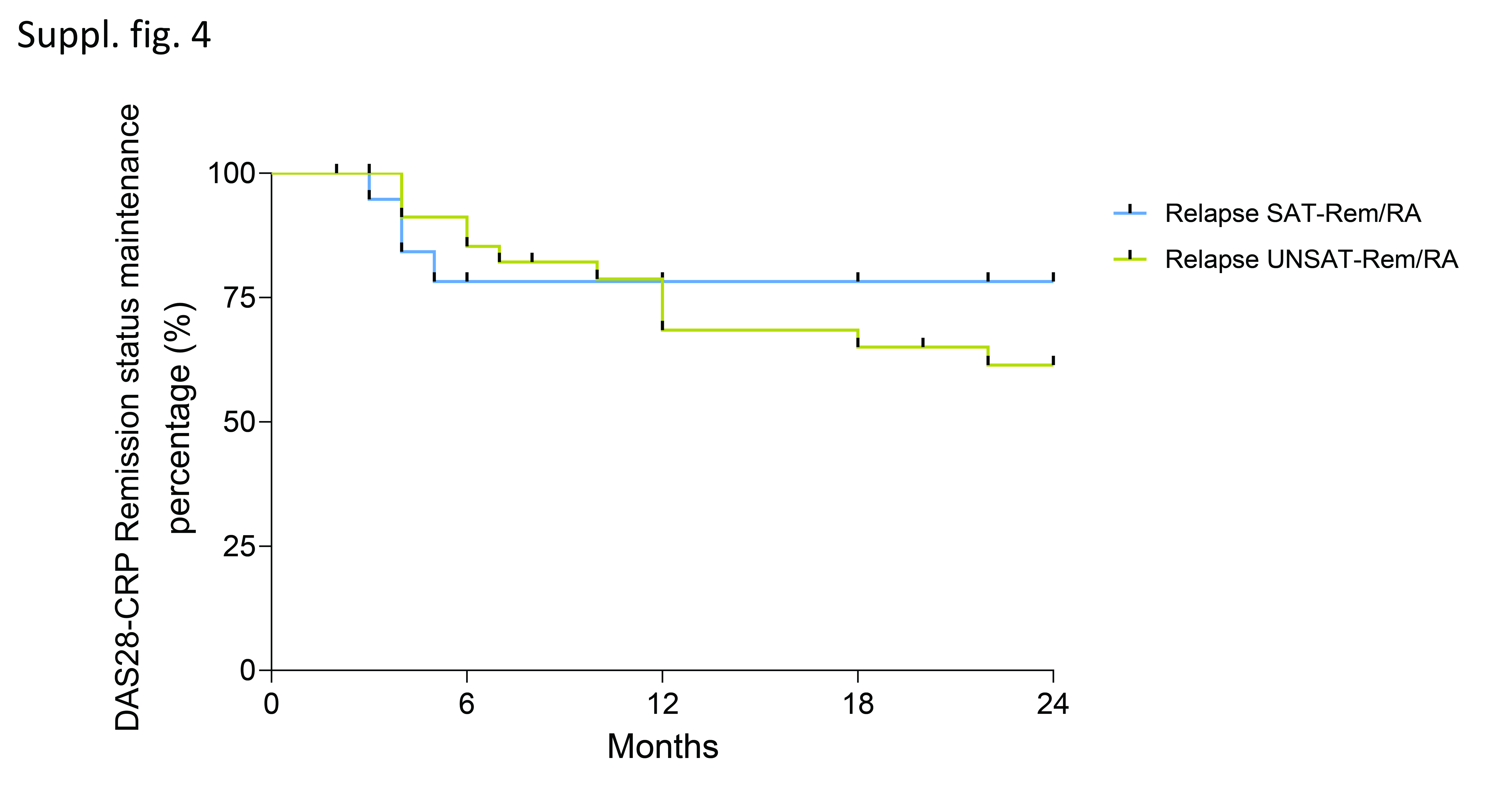

Supplement: Supplementary file 4 — Supplementary Figure 4. DAS28-CRP based remission (Rem/RA) patients survival curve stratified based on RAID score (RAID < 2 (SAT), RAID ≥ 2 (UNSAT)). Log-rank (Mantel-Cox) test to evaluate the difference in flare occurrence between RA subgroup patients. A p-value ≤ 0.05 was considered statistically significant. (JPG 1698 KB) [file 10067_2025_7331_MOESM4_ESM.jpg]
